# Supplementary material for: A New Analysis of Resting State Connectivity and Graph Theory Reveals Distinctive Short-Term Modulations due to Whisker Stimulation in Rats
Source: Front Neurosci. 2018 May 23;12:334. doi: 10.3389/fnins.2018.00334 (PMC5974228; doi:10.3389/fnins.2018.00334)
Supplement: Supplementary file 1 [file Presentation_1.PDF]

Supplementary Material

A new Analysis of Resting State Connectivity and Graph Theory Reveals Distinctive Short-term Modulations due to Whisker Stimulation in Rats

Silke Kreitz, Benito deCelis Alonso, Michael Uder, Andreas Hess*

*** Correspondence:** Andreas Hess: andreas.hess@fau.de

1 Supplementary Methods:

BOLD whisker stimulation data

<u>Preprocessing</u>
Whisker stimulation data were preprocessed individually for each animal using Brainvoyager QX 2.8 (Brain Innovation B.V. Maastricht, The Netherlands). First, data were inter-slice time corrected in an ascending interleaved scan order with cubic spline interpolation followed by a motion correction using rigid registration and resampling with trilinear/sinc interpolation. For optimization of the signal to noise ratio data were 3D smoothed in the spacial domain using a Gaussian filter with FWHM of 2px and subsequently filtered in the temporal domain after linear detrending by high pass filtering with a cut-off of 9 cycles and Gaussian smoothing with FWHM of 4 sec. The preprocessed data of each animal were subjected to a general linear model (GLM) analysis with whisker stimulation as the only predictor. The resulting statistical parametric maps (SPM) were thresholded using FDR (q=0.05) to detect significantly activated voxels per animal.

<u>Data Analysis</u>
Further analysis was performed using the IDL (Exelis Visual Information Solutions Inc., a subsidiary of Harris Corporation, Melbourne, FL, USA) application MagnAn (BioCom, Uttenreuth). The first volume of each animal's functional scan was used as individual anatomical reference. The most unrotated and undisturbed anatomical references was selected by visual inspection to serve as template for the rigid (5 degrees of freedom: 3 for translation, 1 for rotation and 1 for global scaling) registration of the remaining ones. The registered anatomical references were averaged. Afterwards, the same 3D digital rat brain atlas that was used for the analysis of the resting state data was registered (affine, 6 degrees of freedom: 3 for translation and 3 for scaling, no rotation necessary) on the average anatomical template. The sizes of each brain area within the registered digital atlas were determined. Subsequently the registered brain atlas was transformed back to match the individual anatomical references in the animal's native space. These individual brain structure masks were used to determine the number of activated voxels per brain structure and animal.

<u>Statistics</u>
The mean number of voxels per brain structure over all animals was tested to be equal to 0 using a one sided student's t-test. A level of $p < 0.05$ was chosen to be significant. Only mean voxel numbers that differ significantly from 0 were kept, the others were set to 0. Finally, the previously defined

brain structure sizes of the initially registered digital brain atlas were used to calculate the mean activated volume per brain structure in percentual relation to its size.

Network graphs

To obtain representative group correlation maps, we averaged the individual correlation matrices of all subjects for each graph-theoretical approach. These average correlation matrices were transformed into network graphs consisting of vertices (or nodes) and edges. Vertices represent the brain regions, whereas edges between pairs of brain regions indicate their functional connectivity. The topology of a network graph is strongly dependent on the number of represented connections. The connections configuring a graph with a given sparsity were defined by thresholding the associated correlation matrix with a fixed threshold or a fixed number of connections. The former procedure accounts for the overall strength of the matrix and results in different numbers of connections per graph (i.e. different sparsity), the latter leads to different threshold values per matrix but equal sparsity and therefore comparable graph-topology. Here it should be considered, that in undirected graphs (RCCA and SRCC) each connection accounts for two directed connections (MSRA).

ICA co-activation index

To evaluate the correspondence between ICA and each graph-theoretical analysis, we calculated an "ICA co-activation index" as proposed by Rosazza et al. (2012). The z-scores (resulting from the GIFT ICA analysis) of each brain region were averaged and multiplied for each pair of brain regions resulting in a 179x179 product matrix. This procedure was performed for each ICA component separately and the product matrices of all ICA components were summed. Adding a power factor k to the procedure described above emphasizes either the relative weight of intense ($k>1$) or less intense ($k<1$) co-activation. For the calculation of the ICA co-activation matrix we applied the same methodological constraints used to create the graph-theoretical correlation matrices: (1) only positive z-scores were considered and negatives set to 0 and (2) for each subject a separate ICA co-activation matrix was calculated and subsequently averaged to create a group co-activation index.

2 Supplementary Figures and Tables

2.1 Supplementary Figures

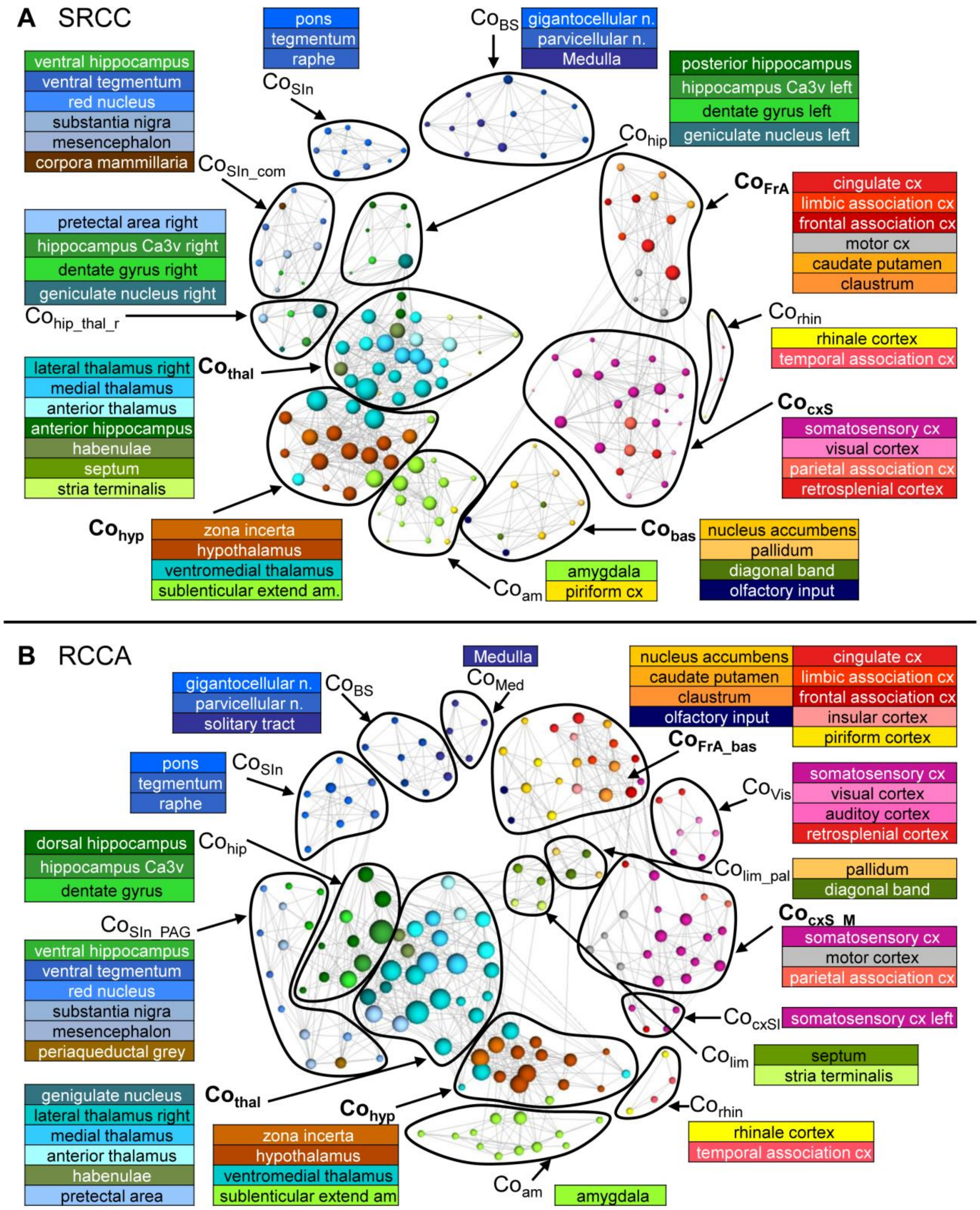

Supplementary Figure 1. Communities of the average resting state network resulting from the SRCC **(A)** and the RCCA **(B)** approach (n=25). Node positions are determined using a forced based algorithm. For better visualization the underlying networks comprise the strongest 895 undirected connections and only communities that contain at least 4 nodes are shown.

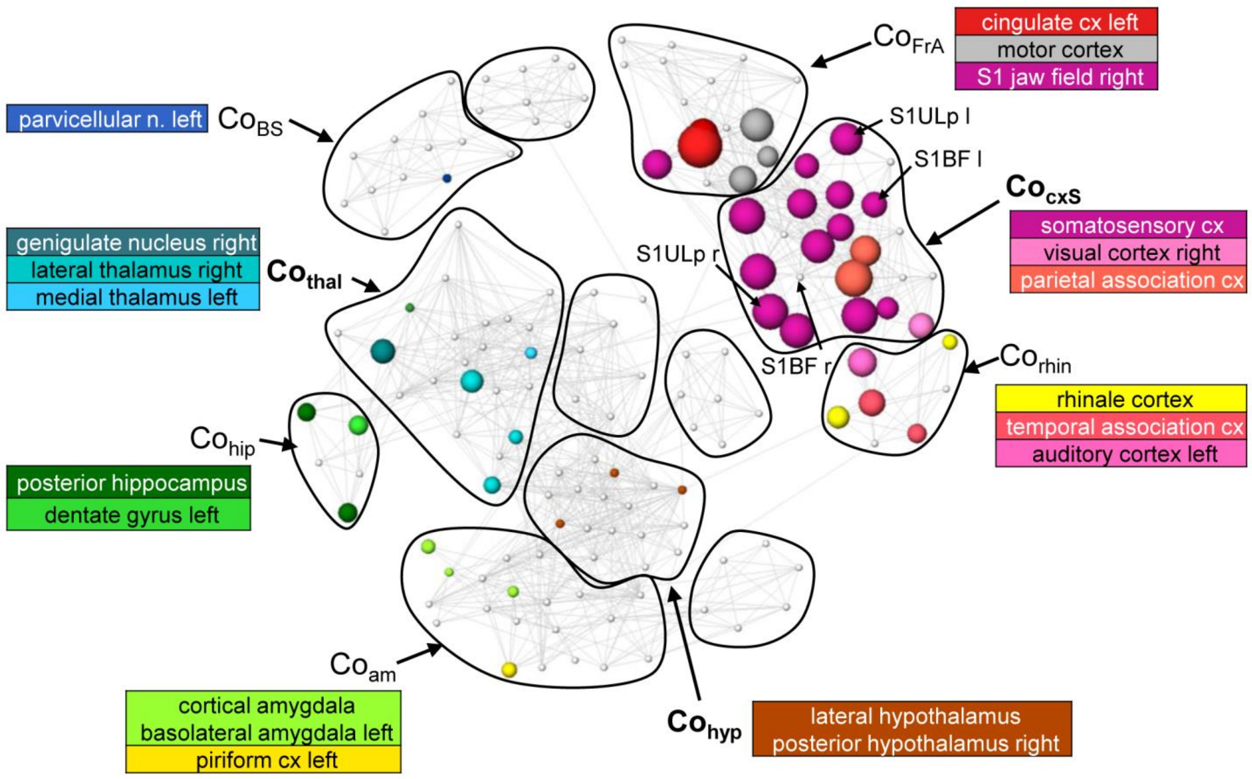

Supplementary Figure 2: Significantly activated brain structures due to whisker stimulation. For better comparability to the modulated resting state connectivity the results are presented using the same visualization scheme and community names as in Fig. 10. Node sizes code the percentual activation volume in relation to the volume of the corresponding brain structure. They range from 3% to 41%. S1BF: primary somatosensory cortex barrel field, S1Ulp: primary somatosensory cortex upper lip field, l: left hemisphere, r: right hemisphere.

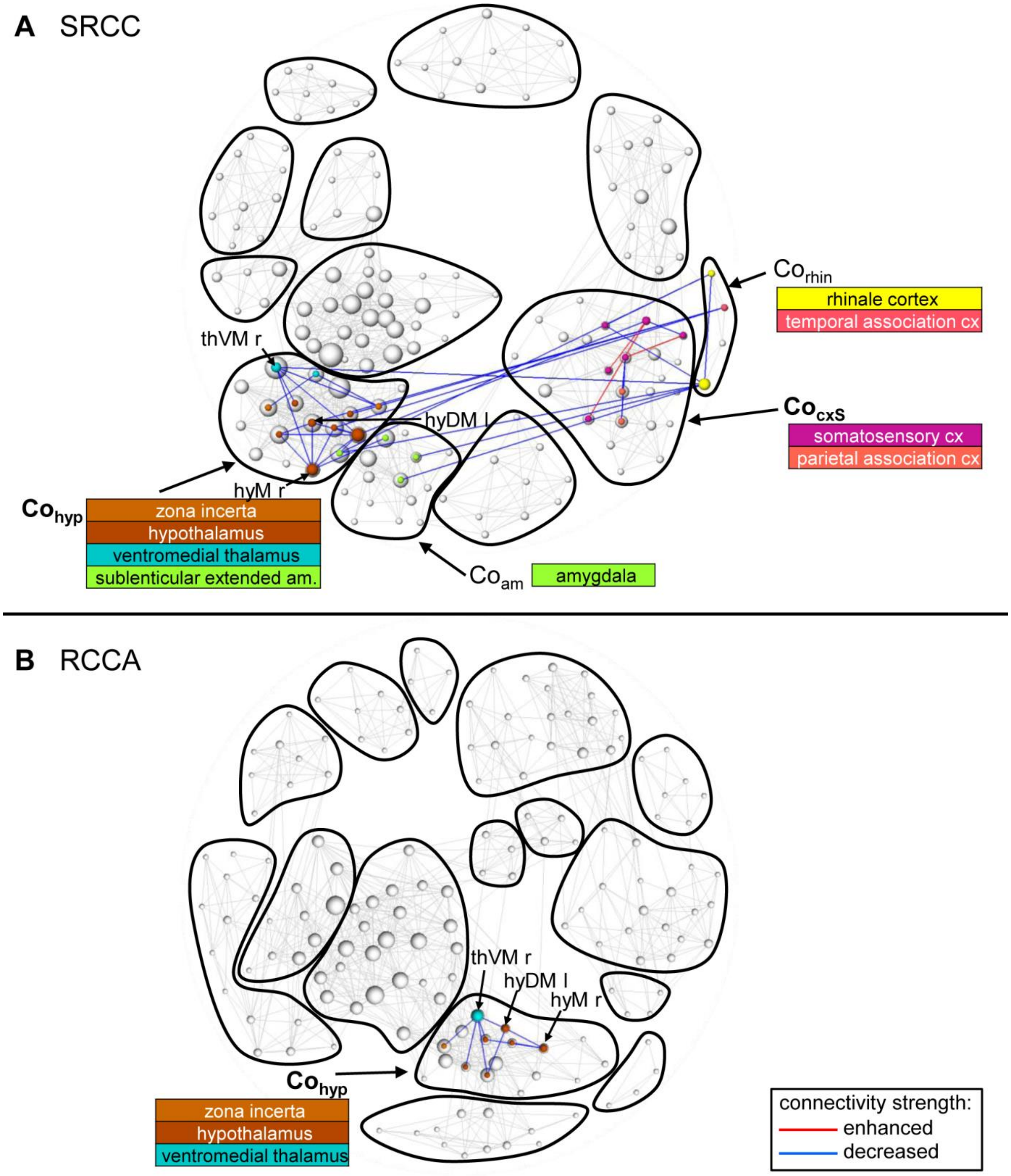

Supplementary Figure 3: Alterations in resting state connectivity due to whisker stimulation. Components of connectivity alterations identified by pNBS using **(A)** RCC (p=0.489, FWE corrected) and **(B)** SRCC (p=0.134, FWE corrected) overlaid on the corresponding resting state networks before whisker stimulation (grey edges and nodes). The visualization scheme is the same as in Fig. S1. hyDM l: left dorsomedial hypothalamus, hyM r: right medial hypothalamus, thVM r: right ventromedial thalamus.

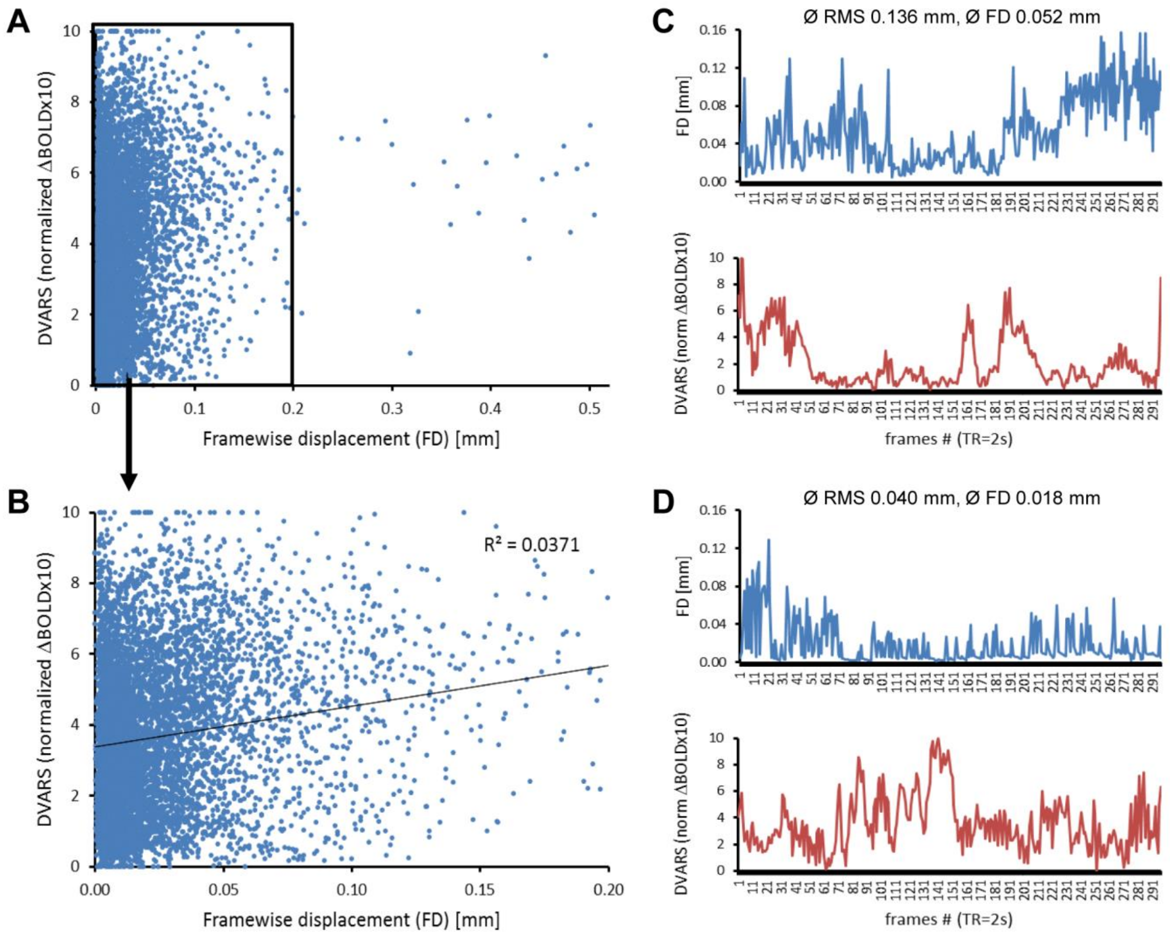

Supplementary Figure 4: Influence of motion on RS BOLD signal change. A) Relation between Frame wise displacement (FD) of head position, calculated as the sum of the absolute values of the 6 translational and rotational realignment parameters, and DVARS, calculated as the whole brain RMS signal change between consecutive frames [according to Power et al. (2012)]. Since original rat fMRI data were not normalized during preprocessing, DVARS are normalized to cover the range of 0 to 10. B) Insert of A), C-D) FD (top) and DVARS (bottom) time-courses of two representative animals, one with high mean RMS movement (C) and one with medium RMS movement (D).
